# Tau Pathology Profile Across a Parietal-Hippocampal Brain Network Is Associated With Spatial Reorientation Learning and Memory Performance in the 3xTg-AD Mouse

**DOI:** 10.3389/fragi.2021.655015

**Published:** 2021-05-19

**Authors:** Alina C. Stimmell, Zishen Xu, Shawn C. Moseley, Sarah D. Cushing, Diana M. Fernandez, Jessica V. Dang, Luis F. Santos-Molina, Rosina A. Anzalone, Carolina L. Garcia-Barbon, Stephany Rodriguez, Jessica R. Dixon, Wei Wu, Aaron A. Wilber

**Affiliations:** ^1^Department of Psychology, Program in Neuroscience, Florida State University, Tallahassee, FL, United States; ^2^Department of Statistics, Florida State University, Tallahassee, FL, United States; ^3^Department of Psychology, University of Florida, Gainesville, FL, United States

**Keywords:** spatial reorientation, hippocampal, parietal, subiculum, retrosplenical cortex, spatial learning and memory, networks, independent component analysis

## Abstract

In early Alzheimer's disease (AD) spatial navigation is one of the first impairments to emerge; however, the precise cause of this impairment is unclear. Previously, we showed that, in a mouse model of tau and amyloid beta (Aβ) aggregation, getting lost represents, at least in part, a failure to use distal cues to get oriented in space and that impaired parietal-hippocampal network level plasticity during sleep may underlie this spatial disorientation. However, the relationship between tau and amyloid beta aggregation in this brain network and impaired spatial orientation has not been assessed. Therefore, we used several approaches, including canonical correlation analysis and independent components analysis tools, to examine the relationship between pathology profile across the parietal-hippocampal brain network and spatial reorientation learning and memory performance. We found that consistent with the exclusive impairment in 3xTg-AD 6-month female mice, only 6-month female mice had an ICA identified pattern of tau pathology across the parietal-hippocampal network that were positively correlated with behavior. Specifically, a higher density of pTau positive cells predicted worse spatial learning and memory. Surprisingly, despite a lack of impairment relative to controls, 3-month female, as well as 6- and 12- month male mice all had patterns of tau pathology across the parietal-hippocampal brain network that are predictive of spatial learning and memory performance. However, the direction of the effect was opposite, a negative correlation, meaning that a higher density of pTau positive cells predicted better performance. Finally, there were not significant group or region differences in M78 density at any of the ages examined and ICA analyses were not able to identify any patterns of 6E10 staining across brain regions that were significant predictors of behavioral performance. Thus, the pattern of pTau staining across the parietal-hippocampal network is a strong predictor of spatial learning and memory performance, even for mice with low levels of tau accumulation and intact spatial re-orientation learning and memory. This suggests that AD may cause spatial disorientation as a result of early tau accumulation in the parietal-hippocampal network.

## Introduction

Alzheimer's disease (AD) is devastating both at the individual and also the societal level (Braak and Braak, 1991; Mitchell et al., 2002; Forner et al., 2017; Mcdade and Bateman, 2017). Individuals with AD have memory, cognitive, and spatial navigational impairments; in fact, getting lost is an early hallmark of AD (Henderson et al., 1989; Billings et al., 2005; Weintraub et al., 2012; Allison et al., 2016; Coughlan et al., 2018). A large body of work, including our own, has found that rodents that recapitulate neurodegenerative features of AD have similar spatial navigation impairments (e.g., Cacucci et al., 2008; Attar et al., 2013; Liu et al., 2013; Marlatt et al., 2013; Webster et al., 2014; Zhao et al., 2014; Stimmell et al., 2019; Cushing et al., 2020). Despite the wealth of research implicating impaired navigation in AD, little is known about the specific changes in the brain that may underlie impaired spatial learning and memory in this devastating disorder. Thus, understanding the neural and environmental conditions that underlie impaired navigation in AD is critical to developing strategies for earlier detection of AD in humans so that interventions can be applied earlier in the progression of the disease.

We previously found that impaired spatial learning and memory is in part a consequence of impaired functional interactions between the parietal cortex and hippocampus (Cushing et al., 2020). A number of previous studies have shown that a variety of spatial learning and memory tasks engage this extended parietal-hippocampal brain network (Morris et al., 1982; Jarrard, 1993; Kolb et al., 1994; Mcnaughton et al., 1995; Aguirre and D'esposito, 1999; Ji and Wilson, 2006; Rogers and Kesner, 2006; Byrne et al., 2007; Nitz, 2012; Whitlock et al., 2012; Pai and Yang, 2013; Sherrill et al., 2013; Wilber et al., 2014, 2017; Maingret et al., 2016; Oess et al., 2017; Tu et al., 2017; Clark et al., 2018b). Finally, there is a large body of evidence that this same brain network is dysfunctional in humans with AD (Jacobs et al., 2012; Morbelli et al., 2012; Wang et al., 2013; Kunz et al., 2015).

Many navigational tasks assume, but do not explicitly test, that mice are using distal cues to navigate the environment. Thus, we have used a spatial learning and memory task that requires the mouse to repeatedly get re-oriented in space using distal cues. The mouse is disoriented after every trial and must use these cues to get re-oriented so they can locate a hidden reward location. The task employed here is likely to tax this same parietal-hippocampal brain network (including the parietal cortex, hippocampus and several structures in between) for getting oriented in space (Mcnaughton et al., 1995; Byrne et al., 2007; Deshmukh and Knierim, 2013; Wilber et al., 2014; Oess et al., 2017; Clark et al., 2018a; Høydal et al., 2019).

Therefore, we set out to take a detailed look at the pathology profile in this brain network and the relationship between this pathology profile and spatial reorientation learning and memory. To do so, we assess the contribution of brain regions from both an individual brain region and also a brain networks perspective using tools originally developed for signal detection but adapted more recently for neuroscience data (Knapp, 1978; Kiviniemi et al., 2003). We use the triple transgenic mouse model (3xTg-AD) that expresses three major genes associated with familial AD (APPSwe, PS1M146V, and tauP301L). The 3xTg-AD mouse expresses plaques and tangles which are distributed in a pattern comparable to that observed in humans (Mesulam, 1999; Oddo et al., 2003).

## Methods

3xTg-AD and age-matched non-transgenic control mice, were grouped housed (2–4/cage) in 12:12 h light/dark cycles until the experiment began. Both 3xTg-AD mice (originally from Dr. LaFerla), and non-transgenic controls of the same background strain as 3xTg-AD mice, were bred in our vivarium. We assessed both young and older male and female mice: 3-month female mice (*n* = 5/genotype; *n* = 10), 6-month mice (*n* = 5/sex/genotype; *n* = 20), and 12-month male mice (*n* = 5/genotype; *n* = 10). We started with 6-month male and female mice. Since females were impaired at spatial reorientation performance at this age and males were not (Stimmell et al., 2019), we then looked at an earlier timepoint in female mice (3-months) to see if they had intact learning at an earlier timepoint, when we expected less tau and Aβ accumulation. Similarly, since 6-month male 3xTg-AD mice were not impaired, we next looked at a later timepoint in male mice (12-months) to see if an impairment developed at a timepoint when we expected more tau and Aβ accumulation. Spatial reorientation training data was presented and genotype was confirmed on all mice previously (Stimmell et al., 2019). The behavior data was used again here for assessing relationships between behavior, and new brain data and analyses. All experimental procedures were carried out in accordance with the NIH Guide for the Care and Use of Laboratory Animals and approved by the Florida State University Animal Care and Use Committee. Experimental procedures were done as previously described (Stimmell et al., 2019; Cushing et al., 2020), but will be described briefly below.

### Pre-training

Mice were water deprived and then trained to alternate between a barrier at the end of a linear track and back for a water reward (*alternation training*). The track can be moved to different starting positions, so the length of the track varied from 1 of 9 randomly selected start locations for both *alternation training* and *spatial reorientation training*. After reaching criteria, mice were scheduled for surgery to implant stimulating electrodes into the medial forebrain bundle (MFB).

### Stimulating Electrode Implantation

Next, mice underwent surgery to implant bilateral stimulating electrodes targeting the left and right MFB (1.9 mm posterior to bregma, 0.8 mm lateral, 4.8 mm below dura) to allow for intracranial stimulation of the medial forebrain bundle (Carlezon Jr and Chartoff, 2007).

### Stimulation Parameters

Following a 1-week recovery period, mice were placed in a custom box with a nose poke port (Med Associates) on one wall. Mice were shaped to automatically trigger brain stimulation with beam breaks by nose poking. A custom MATLAB program delivered one 500 ms brain stimulation reward for each nose poke. Over the course of 1 week, settings were adjusted to achieve maximal response rate and assessed for group differences (which were not present) as described previously (Stimmell et al., 2019; Cushing et al., 2020).

### Spatial Reorientation Training

Once mice were responding maximally, an additional refresher *alternation training* session was conducted, then *spatial reorientation training* commenced (adapted from Rosenzweig et al., 2003; as in Stimmell et al., 2019; Cushing et al., 2020). Throughout the remainder of training and testing, mice shuttled back and forth for a water reward that was delivered in the start box and consumed while the track was moved to the next randomly selected starting location. Next, an unmarked reward zone was added to the task that could automatically trigger a single brain stimulation reward if the mouse remained in the zone for a sufficient period. This zone was fixed in relationship to the room and cues positioned around the periphery of the room. There were no visible markings indicating the reward zone location and olfactory cues were obscured by the moving track. If the mouse remained in the zone for the duration of a delay period (starting at 0.5 s), then a reward was delivered. The delay was fixed for a given day and was increased by 0.5 s (up to 2.5 s) each time the mouse met a criterion as described previously (Stimmell et al., 2019; Cushing et al., 2020). To ensure that mice were performing the task as intended, at the conclusion of training mice underwent two probe tests as described previously (Stimmell et al., 2019; Cushing et al., 2020). Mice were recorded and position was tracked using video tracking software (Neuralynx; 30 Hz frame rate). Velocity data for each position from the tracking data were analyzed using custom Matlab (Mathworks) scripts. We converted these velocity values to *Z*-scores to normalize performance across mice with different running speeds.

### Histology and Imaging

After performing the behavioral task, mice were euthanized by an intraperitoneal injection of Euthasol and then transcardially perfused with 1x phosphate-buffered saline (PBS), followed by 4% paraformaldehyde (PFA) in PBS. Whole heads were post-fixed for 24 h (to allow for easy identification of stimulating electrode tracts targeting the MFB), followed by brain extraction and post-fixing for an additional 24 h. Last, the brain was cryoprotected in a 30% sucrose solution. Frozen sections were cut coronally at a thickness of 40 μm using a sliding microtome and split into six evenly spaced series. Except for 6E10 as noted below, histology was performed on free-floating sections. Following histological processing, whole slides were imaged at 20–40x magnification and stitched together. Histology was performed as described previously (Stimmell et al., 2019; Cushing et al., 2020), briefly:

#### M78

Free floating sections were blocked then incubated in primary antibody anti-MOC78 (monoclonal, rabbit, abcam 205341; an amyloid fibril conformation specific antibody) for two days then washed, followed by secondary antibody anti-rabbit-alexa-488 (goat). To stain for NeuN, sections were then incubated with fluorochrome-labeled primary antibody anti-NeuN-Cy3 (polyclonal, rabbit, Milipore, ABN78C). Sections were mounted with mounting media containing DAPI, then coverslipped and imaged. Tissue processing problems led to loss of M78 material for two mice (one 6-month female mouse and one 6-month male mouse).

#### Phosphorylated Tau

Free floating sections were blocked then incubated in primary antibodies anti-Phosphorylated tau (AT8; monoclonal, mouse, Thermo Scientific; ser202, Thyr205, and possible also ser199; Hanger et al., 2009) and anti-NeuN (polyclonal, rabbit, Milipore) overnight, then washed. Secondary antibodies were anti-mouse-alexa-488 and anti-rabbit-alexa-594 respectively, with incubation for 6 h. Sections were rinsed and mounted onto slides. Tissue processing problems led to loss of pTau material for one 3-month female mouse.

#### Thioflavin S

Free floating sections were blocked then incubated with primary antibody Anti-NeuN overnight, followed by secondary anti-rabbit-alexa-594 for 5 h. Sections were rinsed then immersed in a 1% Thioflavin S solution (Sigma) for 9 min, rinsed in dH_2_O, destained in 70% Ethanol for 5 min, rinsed in dH_2_O, and then transferred to TBS before mounting onto slides.

#### Parvalbumin

Free floating sections were quenched in 0.3% H_2_O_2_ for 25 min, then blocked in 5% goat serum 90 min. They were then incubated with primary antibody mouse anti-parvalbumin (Sigma Aldrich) for 2 days followed by a biotinylated goat anti-mouse antibody (Sigma Aldrich) for 90 min. Following this, solutions A and B from the standard Vectastain ABC kit (Vector Laboratories) 1:500 for 1 h. Staining was developed using a DAB (3,3′-Diaminobenzidine tetrahydrochloride hydrate; Sigma Aldrich) solution. The sections were then mounted onto slides. After air drying, slides were dehydrated in increasing concentration of alcohol, cleared with Hemo-De and coverslipped with Fisher Chemical Permount™ Mounting Medium.

#### 6E10

Free floating Sections were first mounted on slides before being incubated in 4% PFA for 4 min, then 70% formic acid for 8–15 min. Next, sections were blocked and incubated in primary antibodies anti-β-amyloid 1–16 (mouse, clone 6E10, Biolegend) and anti-NeuN for 2 days. Next, sections were washed then incubated in secondary antibodies anti-mouse-alexa-488 and anti-rabbit-alexa-594 for 5–6 h. Finally, sections were washed and coverslipped with a mounting medium containing DAPI.

### Region of Interest Analyses

**Figure 1:**
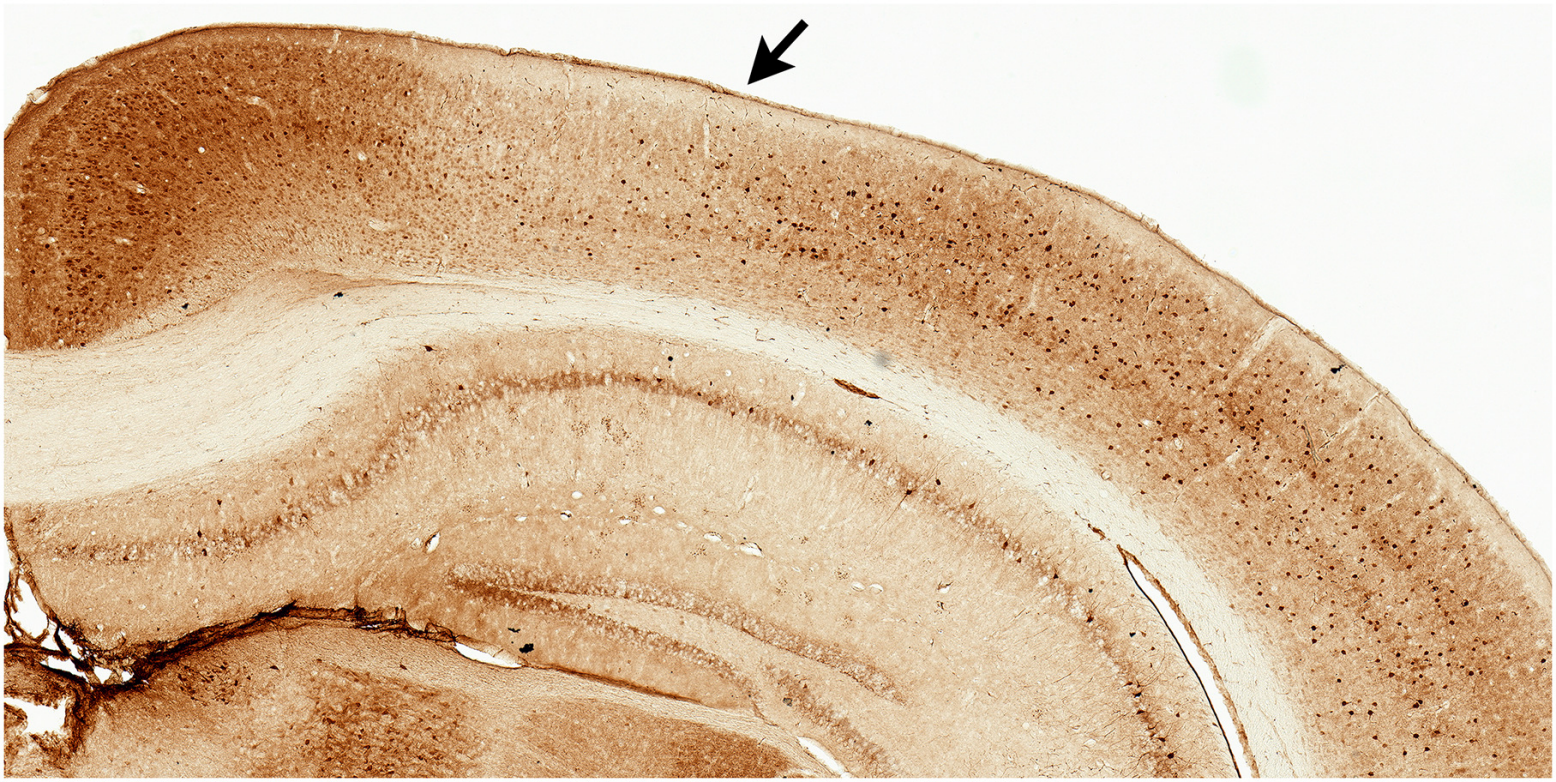
Parvalbumin stained tissue for region of interest analyses. Example of facilitated identification of a region of interest, parietal cortex (PC), using adjacent parvalbumin stained tissue. PC (arrow) can be readily identified by the emergence of layer IV (medial boundary) and ending at the lateral border where parvalbumin staining becomes more robust.

The density of cells positive for M78, 6E10, and phosphorylated tau intracellular pathology was estimated for four general regions of interest (retrosplenial cortex, CA1 field of the hippocampus, subiculum, and parietal cortex) which were further subdivided as follows. Dorsal CA1 (dCA1) was defined as all sections rostral to a point 2.55 mm posterior to bregma. Ventral CA1 (vCA1) was considered all sections caudal to that same point. Retrosplenial cortex and subiculum were subdivided into dorsal (RSPd and dSub), and ventral (RSPv and vSub). Thus, we examined seven regions in total. Next, an outline was manually drawn around each region of interest (ROI) for each section containing that ROI in a 1:12 evenly spaced series through the entire brain by an experimenter that was blind to group using the manual selection tool in ImageJ (Fiji). ROIs were based on regional boundaries and cytoarchitectural differences in adjacent parvalbumin stained tissue as described previously (Figure 1; Stimmell et al., 2019). The number of M78, 6E10, and pTau labeled cells was then expressed as the proportion per unit of area. Area calculations were done in pixels and converted to mm for illustrative purposes. Manual counts were performed on 10x equivalent images extracted from higher resolution scans. Finally, to ensure that slight differences in counting across raters did not produce spurious effects across groups or ROIs, count densities were converted to *Z*-scores by calculating the mean and SD across all four groups of 3xTg-AD mice (3- and 6- month female mice and 6- and 12- month male mice) and then converting each density value to a *Z*-score. Thus, *Z*-scored densities represent the relative increase or decrease from the mean across all four groups. Statistical comparisons of brain data between groups were performed using two-way repeated measures ANOVAs (group × ROI), followed by post-testing corrected for repeated testing when appropriate (e.g., following interactions). For all statistical analyses, *p* < 0.05 was considered significant after correcting for repeated testing (e.g., after a Bonferroni correction for multiple comparisons was applied). Bonferroni corrected critical values are only shown for Bonferroni corrections that shift an uncorrected significant *p*-value to non-significant and not when the statistical result remains significant after Bonferroni correction. Statistical analyses were performed using the Matlab Statistics toolboxes (Berens, 2009) and Graphpad.

### Canonical Correlation Analyses

In order to understand the influence of pathology patterns across brain regions or across markers on spatial reorientation learning and memory we performed Canonical Correlation Analysis (CCA). CCA is a method for finding the linear relation between two groups of variables (Hotelling, 1992). Suppose *X* ∈ ℝ^*n*×*p*^ and *Y* ∈ ℝ^*n*×*q*^ are two groups of variables with *n* observations, where *X* contains *p* features and *Y* has *q* features. The CCA finds the optimal weights *a* ∈ ℝ^*p*×1^ and *b* ∈ ℝ^*q*×1^ such that the linear combination *Xa* and *Yb* can achieve the largest absolute correlation. If *q* = 1, then *b* is a real value and |*Corr*(*Xa, Yb*)| = |*Corr*(*Xa, Y*)|. In this way, the weight *a* can indicate the contribution of the features in *X* to the linear association between *X* and *Y*. We performed one CCA analysis for each pathology marker (6E10, M78, pTau) collapsed across group (Male, Female, Age). The accuracy of the identified profile was tested by performing correlations between the weighted brain region profile and spatial reorientation performance. Spatial reorientation performance was defined as the mean *Z*-scored speed during the approach to the hidden reward zone for two reward difficulty delays: 1.5 and 2.0 s. This behavioral data was selected for the correlations because we found significant group differences with these reward difficulty delays in our previous paper (Stimmell et al., 2019).

### Independent Components Analyses

Finally, we asked if brain networks could be identified independent of the behavioral data by looking for common features across animals and then asking if any of these features were predictive of behavior. To do this, we used independent components analysis (ICA) to look for common features across animals. ICA was designed for finding a hidden independent source signals in data (Hérault and Ans, 1984; Comon, 1994). For example, suppose *X* ∈ ℝ^*p*×*n*^ with *n* observations and *p* features. ICA assumes the data is a linear combination of potential independent sources. In other words, *X* = *AS* where *S* ∈ ℝ^*r*×*n*^ denotes the hidden sources with *r* independent features and *A* ∈ ℝ^*p*×*r*^ is the linear combination weight matrix. The ICA algorithm will find both *S* and *A* based on *X* only. There are many algorithms to achieve the goal of the ICA. In this paper, we adopt the maximum likelihood approach (Dinh Tuan and Garat, 1997). Here, we used ICA data to identify common features across animals for the pathology profile across brain regions, and then we performed correlations between the weighted ICA brain data profile and spatial reorientation performance for the 1.5 and 2.0 s reward delays. We performed one ICA analysis for each group separately (3- and 6-month female, and 6- and 12-month male). Within each group, we used all possible numbers of sources from 1 to 7 (the number of brain regions). Using an approach that accounts for the increased number of tests with larger numbers of sources, we developed a custom random shuffling method to set critical values for each data set in order to test significance. Specifically, we randomly permuted the rows of the brain pathology data. For each shuffle, we performed ICA under the same settings to obtain the sources and the corresponding correlation. In this way, the empirical distribution of the shuffled correlation can be computed. As we only focus on the correlation with largest absolute value in the original ICA output, we also choose only the largest absolute correlation in each shuffle to make the distributions consistent. If the original correlation meets or exceeds the *p* = 0.05 critical *p*-value of the shuffled correlation distribution, then we can reject the null hypothesis and conclude that there is a significant linear relationship between the brain and behavior data.

## Results

### Female 6-Month 3xTg-AD Mice Have More Dorsal Hippocampal Aβ Positive Cells Than 6-Month Males, While 12-Month Males Have the Highest 6E10 Positive Cell Density Regardless of Brain Region

#### 6E10

**Figure 2:**
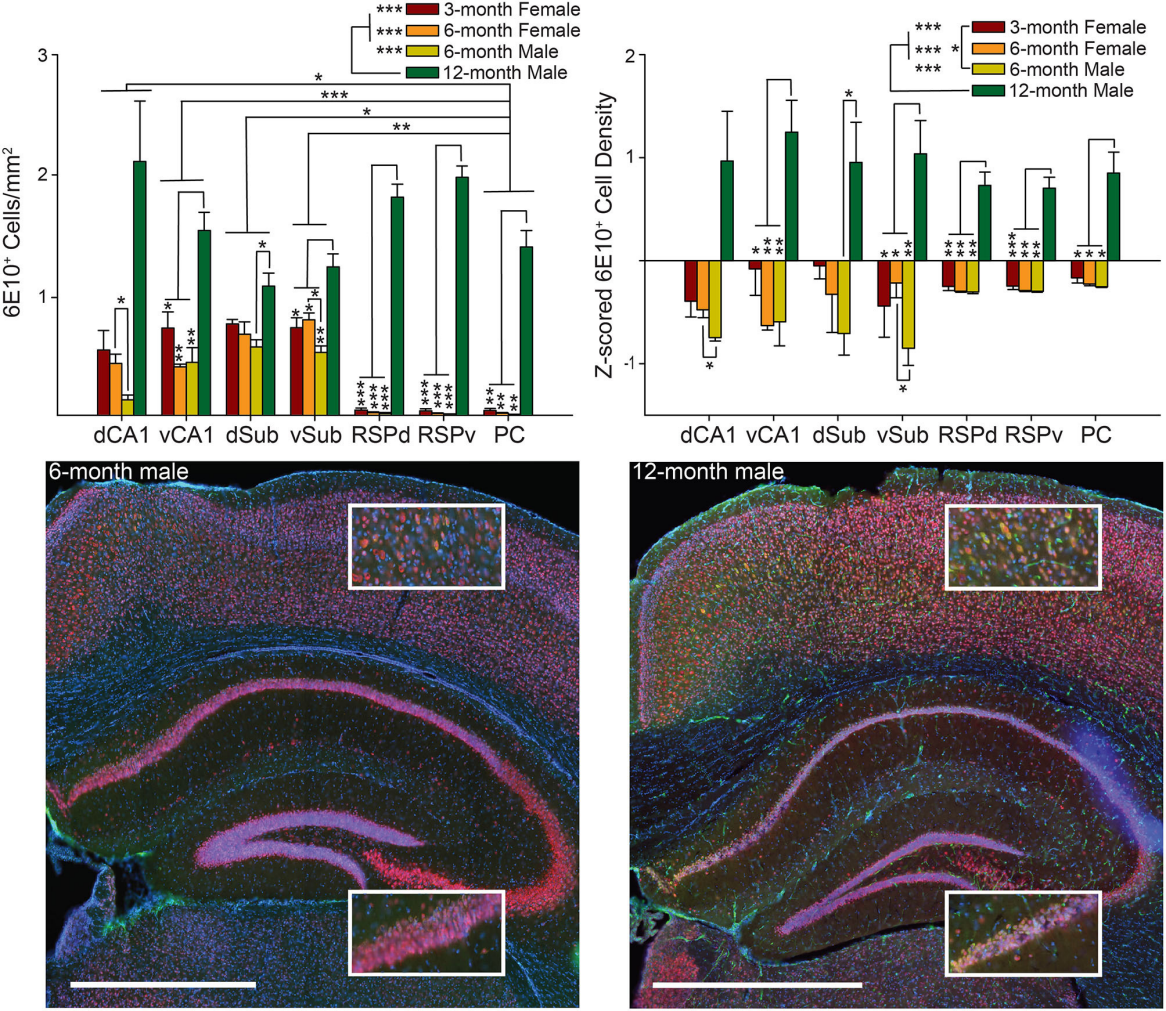
Male 6-month 3xTg-AD mice have a lower 6E10 positive cell density than 6-month females, while 12-month males have the highest 6E10 positive cell density. *Top*. 6E10 positive cell density (*left*) and *Z*-scored density (*right*) for each of the seven brain regions and four groups of mice. *Bottom*. Representative images showing 6E10 staining (green) in Dorsal Retrosplenial Cortex (RSPd; Top Insets) and Dorsal CA1 (dCA1; Bottom Insets) for 6-month (*left*) and 12-month (*right*) male 3xTg-AD mice. Sections were also stained with NeuN (red) and DAPI (blue). Ventral CA1 (vCA1), Dorsal Subiculum (dSub), Ventral Subiculum (vSub), Ventral Retrosplenial Cortex (RSPv), and Parietal Cortex (PC). *p < 0.05, **p < 0.01, ***p < 0.001. Scale bar = 1 mm.

The raw 6E10 data from some brain regions (CA1, RSP, and PC but not Sub) for the 6-month male and female groups (but not the 3-month females or 12-month males) was published previously (Stimmell et al., 2019). When we examined the histology from 3xTg-AD mice at two timepoints for female mice (3- and 6-month groups) and male mice (6- and 12-month groups), we found that Aβ, as measured by the density of 6E10 (Aβ 1-16 specific antibody) positive cells, significantly varied across brain region as a function of group [group × brain region interaction: *F*_(18, 96)_ = 8.81, *p* < 0.0001; Figure 2 *Top Left* and *Bottom*; also main effect of group: *F*_(3, 16)_ = 73.41, *p* < 0.0001 and brain region, *F*_(2, 25)_ = 12.13, *p* < 0.001]. Next, in order to ensure that variability across raters did not contribute to observed effects, we *Z*-scored the data within rater across the four groups, so that the *Z*-scores represent the variance around the mean of the four groups. Again, we found that 6E10 pathology varied significantly across groups [group × brain region interaction: *F*_(18, 96)_ = 1.82, *p* < 0.05; Figure 2 *Top Right*; also main effect of group: *F*_(3, 16)_ = 31.33, *p* < 0.0001, and, since this method removes variability across brain regions, there was no effect of brain region: *F*_(3, 52)_ = 0.89, *p* = 0.50].

To find the source of the interaction, we assessed all pairwise combinations of the four groups for each brain region using the Tukey multiple comparisons test. The pattern of results was identical, though the magnitude of some effects differed, so, for simplicity, combined results from both *Z*-scored and raw density measures are summarized together. Note, the similarity in across group effects for *Z*-scored versus raw density data suggests that across rater differences did not strongly influence the results. We found that 12-month male mice had significantly higher density of 6E10 positive cells than all other groups for most brain regions (RSP, PC, vCA1, vSub: *ps* < 0.05), but not dCA1 and dSub. For dCA1, 12-month males did not differ from any other group (*ps* > 0.05); however, 6-month females had significantly higher density of 6E10 positive cells than 6-month male mice (*ps* < 0.05). For vSub, 6-month females again had significantly higher density of 6E10 positive cells than 6-month male mice (*p* < 0.05). Finally, for dSub, 12-month males only had significantly higher 6E10 cell density than 6-month males (*p* < 0.05), but there were no other significant differences (*ps* > 0.08). Male and female 6-month mice were age matched at the beginning of the experiment and did not differ significantly in age at perfusion at the end of the experiment, as previously reported (Stimmell et al., 2019). No other pairwise comparisons were significant (*ps* > 0.19).

Next, to find the source of the variability underlying the main effect of group we conducted Tukey multiple comparisons for all pairwise group combinations and found that, for both raw and *Z*-scored density data, 12-month males had significantly higher 6E10 positive cell density than all other groups (*ps* < 0.0001). For *Z*-scored density data, 3-month females also had significantly more pathology than 6-month males (*ps* < 0.05). No other group combinations were significantly different (*ps* > 0.24). Finally, to find the source of the variability underlying the brain region main effect for the raw 6E10 density data, we performed Tukey multiple comparisons tests for all pairwise brain region combinations. We found the PC had significantly lower 6E10 positive cell density than both subregions of Sub and CA1 (*ps* < 0.05); however, there were no significant differences between any remaining brain regions (ps > 0.06).

#### M78

**Figure 3:**
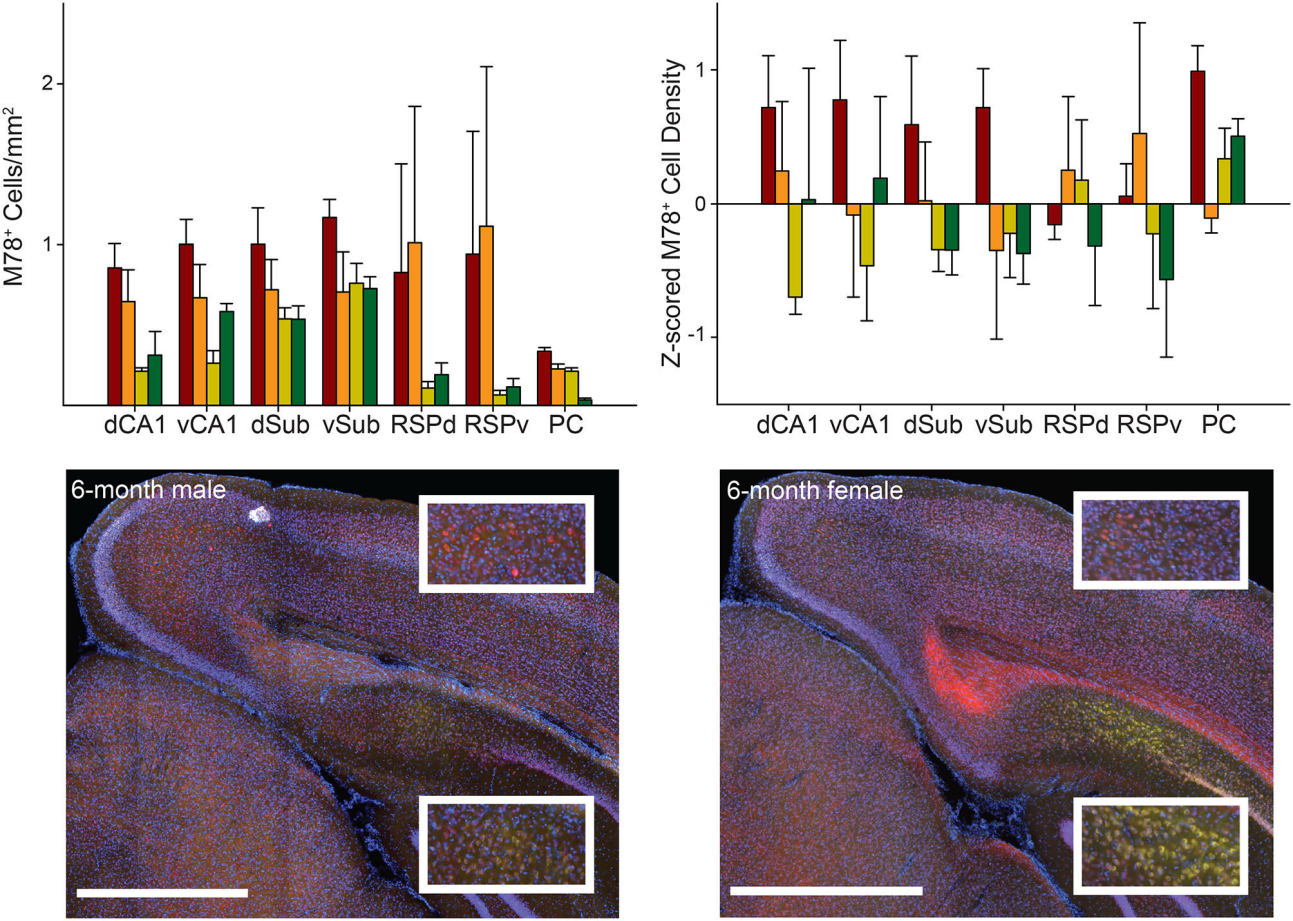
M78 staining did not significantly vary across groups or brain regions. *Top*. M78 positive cell density (*left*) and Z-scored density (*right*) for each of the seven brain regions and four groups of mice. *Bottom*. Representative images showing M78 staining (green) in RSPd (Top Insets) and dCA1 (Bottom Insets) for 6-month male (*left*) and female (*right*) 3xTg-AD mice. Sections were also stained with NeuN (red) and DAPI (blue). Ventral CA1 (vCA1), Dorsal Subiculum (dSub), Ventral Subiculum (vSub), Ventral Retrosplenial Cortex (RSPv), and Parietal Cortex (PC). Scale bar = 1 mm.

The pattern across groups was generally similar for M78 (an amyloid fibril conformation specific antibody; Pensalfini et al., 2014) compared to 6E10 except that male mice, particularly 12-month male mice, had much less M78 positive cell density than 6E10 density. Potentially as a result of this difference with M78 versus 6E10 data, raw and *Z*-scored M78 positive cell density did not significantly vary across brain region as a function of group [group × brain region interaction: *F*s_(18, 84)_ < 1.67, *ps* > 0.06; Figure 3; also no main effect of group: *F*s_(3, 14)_ < 2.56, *ps* > 0.09 and brain region, *F*s_(1−2, 15−30)_ < 1.60, *ps* < 0.23].

### 12-Month Male 3xTg-AD Mice Have the Highest Density of pTau Positive Cells

**Figure 4:**
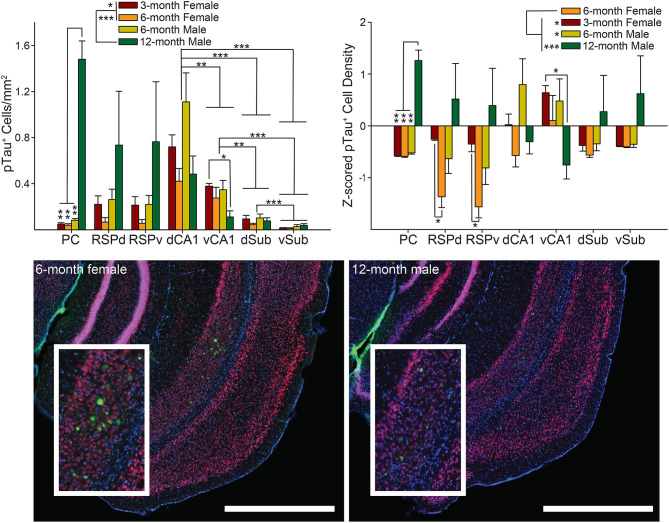
Male 6-month 3xTg-AD mice have the highest density of intracellular tau positive cell density. *Top*. Tau positive cell density (*left*) and *Z*-scored density (*right*) for each of the seven brain regions and four groups of mice. *Bottom*. Representative images showing pTau staining (green) in vSub (Insets) for 6-month female (*left*) and 12-month male (*right*) 3xTg-AD mice. Interestingly, despite the low density of pTau positive cells, staining in 6-month female mice was much brighter in vSub than any other brain region; however, staining was not bright in 12-month males. Sections were also stained with NeuN (red) and DAPI (blue). Dorsal CA1 (dCA1), Ventral CA1 (vCA1), Dorsal Subiculum (dSub), Ventral Subiculum (vSub), Dorsal Retrosplenial Cortex (RSPd), Ventral Retrosplenial Cortex (RSPv), and Parietal Cortex (PC). *p < 0.05, **p < 0.01, ***p < 0.001. Scale bar = 1 mm.

The raw density of pTau positive cells significantly varied across brain region as a function of group [group × brain region interaction: *F*_(18, 90)_ = 4.50, *p* < 0.0001; Figure 4 *Top Left* and *Bottom*; also main effect of group: *F*_(3, 15)_ = 4.17, *p* < 0.05 and brain region, *F*_(1.5, 22)_ = 8.18, *p* < 0.01]. Next, in order to ensure that variability across raters did not contribute to observed effects, we *Z*-scored the data within rater across the four groups, so that the *Z*-scores represent the variance around the mean of the four groups. Again, we found that pTau pathology varied significantly across groups as a function of brain region [group × brain region interaction: *F*_(18, 90)_ = 2.71, *p* < 0.01; Figure 4 *Top Right*; also main effect of group: *F*_(3, 15)_ = 4.14, *p* < 0.05, and since this method removes variability across brain regions, no effect of brain region: *F*_(2.4, 36.7)_ = 2.05, *p* = 0.13].

To find the source of the interaction, we assessed all pairwise combinations of the four groups for each brain region using the Tukey multiple comparisons test. The pattern of results was identical, though the magnitude of some effects differed; so, for simplicity, results from both *Z*-scored and raw density measures are summarized together. Again, the similarity in across group effects for *Z*-scored vs. raw density data suggests that across rater differences did not strongly influence the results. For raw and *Z*-scored density data, we found that 12-month male mice had a higher density of pTau positive cells than all other groups for PC (*ps* < 0.01) and 12-month male mice had a higher pTau positive cell density than 3-month female mice for vCA1 (ps = 0.02). For PC and vCA1 no other pairwise comparisons were significant (*ps* > 0.15). For *Z*-scored, but not raw, densities in both subfields of RSC, 6-month females had significantly more pTau positive cells than 3-month females (*ps* < 0.02). There were no significant group differences for the remaining brain regions (RSP, dCA1, and Sub: *ps* > 0.16).

Next, to find the source of the variability underlying the main effect of group, we conducted Tukey multiple comparisons for all pairwise group combinations and found that, for raw density data, 12-month males had significantly higher pTau positive cell density than 3- and 6-month female mice (*ps* < 0.05). For *Z*-scored densities, 6-month females had less pathology than all other groups (*ps* < 0.05). No other group combinations were significantly different (*ps* > 0.05).

Finally, to find the source of the variability underlying the brain region main effect for the raw 6E10 density data, we performed Tukey multiple comparisons tests for all pairwise brain region combinations. We found that dCA1 had significantly higher pTau positive cell density than both subregions of Sub and vCA1 (*ps* < 0.01). In addition, vCA1 had significantly higher pTau density than both subregions of Sub (*ps* < 0.01), and dSub had higher density than vSub (*p* = 0.0003). There were no significant differences between any remaining brain regions (*ps* > 0.15).

### pTau Positive Cell Density Is Positively Correlated With 6E10 Density; While Both pTau and 6E10 Are Negatively Correlated With M78 Positive Cell Density

Finally, we examined the relationship between the density of positive cells for each pairwise combination of the three markers of pathology (pTau, 6E10, and M78) collapsed across group for individual brain regions and collapsed across brain region. For raw density data, when we collapsed across brain region, even after correcting for repeated testing, pTau positive cell density remained positively correlated with 6E10 positive cell density (*r* = 0.24, *p* = 0.006), but no other pairwise stain combinations were significantly correlated (*rs* > −0.15, *ps* < 0.10). Similarly, *Z*-scored pTau density was significantly negatively correlated with M78 positive cell density (*r* = −0.19, *p* = 0.04), but no other pairwise stain combinations were significantly correlated (*rs* < 0.08, *ps* > 0.34).

**Figure 5:**
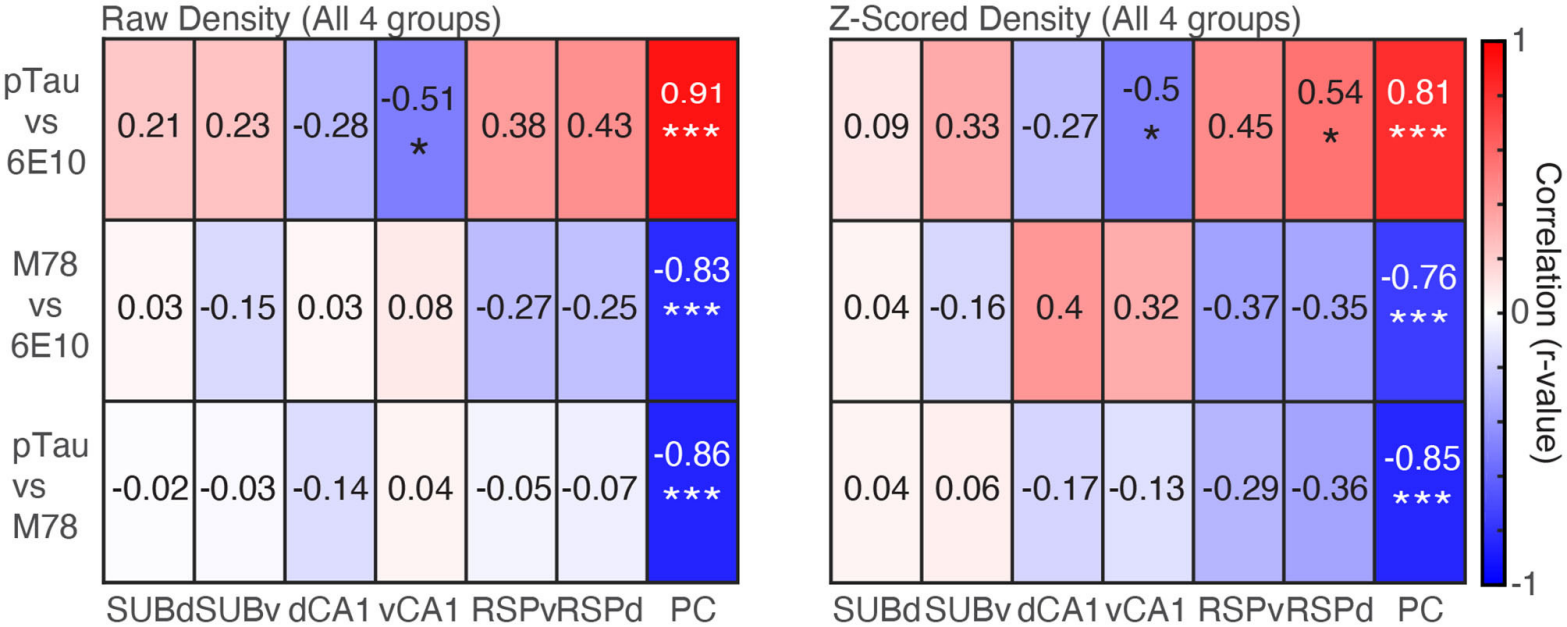
Densities of pTau and 6E10 are positively correlated, while M78 is negatively correlated with both pTau and 6E10. *Left*. Raw density correlations for pairwise combinations of each marker of pathology (pTau, M78 and 6E10) for each of the seven brain regions. *Right*. Same as Left, but for *Z*-scored density data. *p < 0.05, ***p < 0.001.

Next, when we performed the same analysis separately for each of the seven brain regions, for raw density data, PC pTau positive cell density was positively correlated with 6E10 (Figure 5 *Left*; *r* = 0.91, *p* < 0.0001); while both pTau and 6E10 were negatively correlated with M78 (*rs* > −0.83, *ps* < 0.0001). Last, vCA1 pTau was significantly negatively correlated with 6E10 positive cell density (*r* = −0.51, *p* = 0.03). No other brain regions were significantly correlated (*rs* < 0.43, *ps* > 0.06). A similar pattern was apparent for *Z*-scored density; PC pTau positive cell density was positively correlated with 6E10 (Figure 5 *Right*; *r* = 0.81, *p* < 0.0001), while both pTau and 6E10 were negatively correlated with M78 (*rs* > −0.76, *ps* < 0.001). Last, RSPd pTau was also significantly positively correlated with 6E10 positive cell density (*r* = 0.54, *p* = 0.02), while vCA1 pTau was significantly negatively correlated with 6E10 positive cell density (*r* = −0.50, *p* = 0.03). No other brain regions were significantly correlated (*rs* < 0.45, *ps* > 0.06). Thus, in general, pTau was positively correlated with 6E10 while M78 was negatively correlated with 6E10 and pTau; though these correlations may be largely driven by pathology positive cell densities in PC. Interestingly, hippocampal correlations were frequently in the opposite direction as cross-marker correlations for all other brain regions.

### CCA Analyses Revealed a pTau and 6E10 Profile for All Groups of 3xTg-AD Mice That Predicts Spatial Reorientation Learning and Memory

In the spatial re-orientation task, mice slow as they approach the reward zone in order to stop in the reward zone for the required amount of time (reward delay) to obtain a brain stimulation reward (Stimmell et al., 2019). To capture spatial re-orientation ability in 3xTg-AD mice, we isolated velocity data (*Z*-scored) for a short distance just before the front edge of the reward zone. We previously found that *Z*-scored velocity allows for comparison of performance across reward delays (i.e., across the range of task difficulty), and avoids other potential confounds as described previously (Stimmell et al., 2019). The mean *Z*-scored velocity in front of the reward zone was calculated for each mouse for the 1.5 and 2.0 s reward delays and was used for all correlations reported here. This behavioral data was selected for the correlations because we found significant group differences with these reward difficulty delays in our previous study (Stimmell et al., 2019). First, we performed linear regressions separately for each stain (pTau, 6E10, and M78) between 1.5 and 2.0 s reward delay data and each of the seven brain regions with a Bonferroni repeated test significance correction. *Z*-scored densities did not differ significantly across brain region and produced very similar patterns across groups compared to raw densities (Figures 2–4), therefore raw densities were used for all subsequent analyses. Further, because there were no significant group or region differences for M78 data, M78 densities were not used for subsequent analyses.

#### pTau

**Figure 6:**
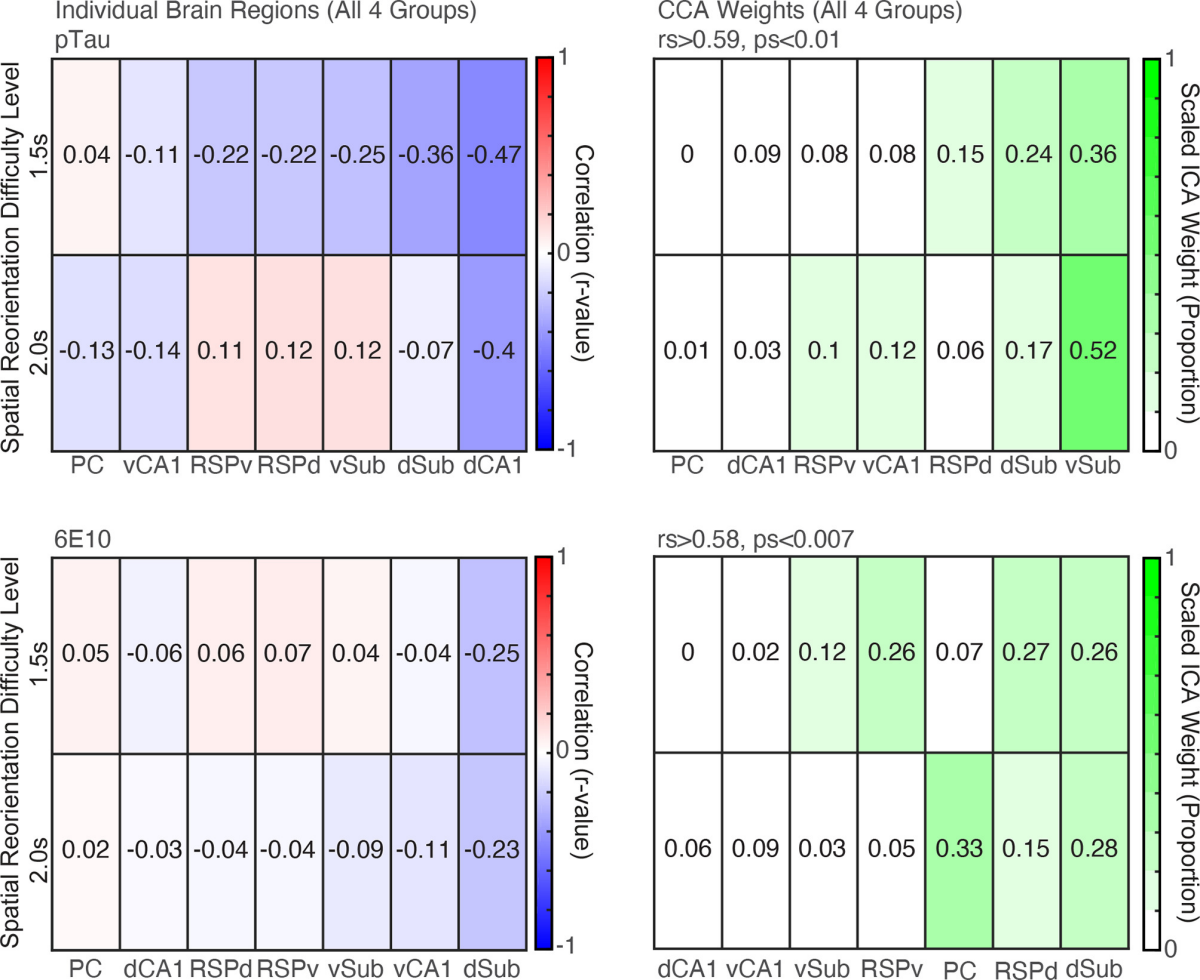
CCA analyses revealed profiles of pTau and 6E10 densities that were significant predictors of subsequent behavior. *Top*. *Left*. Correlations between raw pTau density for individual brain region and spatial reorientation performance for the 1.5 and 2.0 s difficulty levels for all four groups combined (3- and 6- month female, 6- and 12- month male). *Right*. Brain region weights (expressed as a proportion) identified by Canonical Correlation Analysis (CCA) for the linear combination that was significantly correlated with spatial reorientation performance for the 1.5 and 2.0 s difficulty levels for all four groups. *Bottom*. *Left* and *Right*. Same as *Top* Left and *Right*, except for 6E10 density.

Individual brain region correlations were small, and none were statistically significant (Figure 6, *Top Left*; *rs* > −0.47, *ps* > 0.04. adjusted critical value 0.007). However, there was a significant correlation between the weighted brain profiles identified by CCA analysis and behavior for both difficulty levels (Figure 6, *Top Right*; *rs* > 0.59, *ps* < 0.007). The Sub received the highest CCA weight.

#### 6E10

6E10 correlations were also small and none were statistically significant (Figure 6, *Bottom Left*; *rs* > −0.25, *ps* > 0.28. adjusted critical value 0.007). However, there was a significant correlation between the weighted brain profiles identified by CCA analysis and behavior for both difficulty levels (Figure 6, *Bottom Right*; *rs* > 0.58, *ps* > 0.007). The dSub and dRSC received the highest weights in the linear combination of the seven brain regions that produced this significant correlation with behavior.

### ICA Analyses Revealed That 3xTg-AD Mice From Each Group Have a pTau Positive Cell Density Profile That Is a Significant Predictor of Spatial Re-orientation Performance

#### 6-Month Female Mice Individual Brain Region Correlations With Spatial Learning and Memory

**Figure 7:**
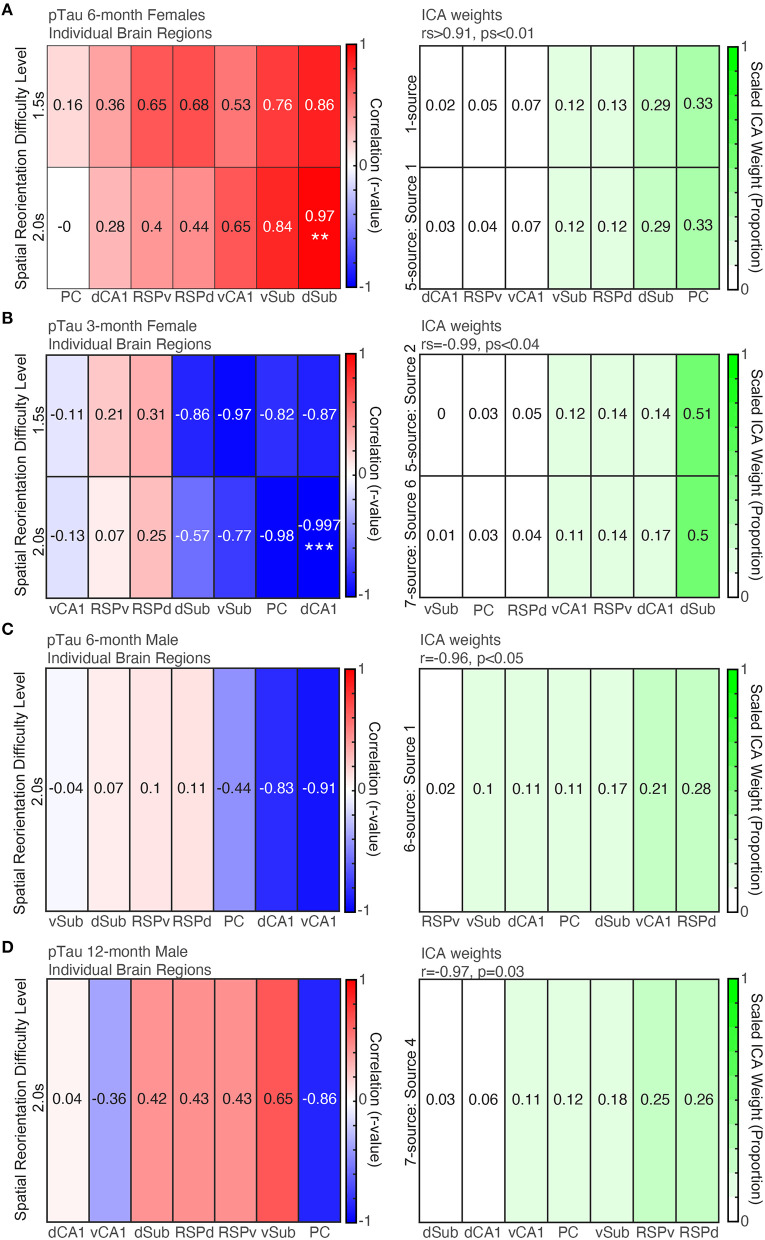
More pTau pathology is predictive of spatial reorientation performance, but only for 6-month female mice. *Left*. Correlations between pTau raw density for individual brain regions and spatial reorientation performance for the 1.5 and 2.0 s difficulty levels for 6-month female (**A**) and male (**B**) mice, 3-month female mice (**C**), and 12-month male mice (**D**). *Right*. Brain region weights (expressed as a proportion) identified by Independent Components Analysis (ICA) for the linear combination that was significantly correlated with spatial reorientation performance for the 1.5 and 2.0 s difficulty levels for 6-month female (**A**) and male (**B**) mice, 3-month female mice (**C**), and 12-month male mice (**D**). **p < 0.01, ***p < 0.001.

Before examining ICA results to assess patterns across brain regions in 6-month female mice, we first performed individual correlations between pTau positive cell density for each brain region and spatial re-orientation performance for the 1.5 and 2.0 s difficulty levels. Consistent with previous work linking dorsal structures to spatial navigation, a dorsal structure was the only individual brain region to be significantly correlated with behavior. Higher density of pTau positive cells in dSub was positively associated with poorer spatial reorientation performance for the 2.0 s difficulty level (less slowing in the reward zone; *r* = 0.97, *p* = 0.005; Figure 7A, *Left*) but not the 1.5 s difficulty level (*r* = 0.86, *p* = 0.06). There was a mixture of dorsal and ventral regions with some large (e.g., vSub, vCA1, and RSPd) but non-significant correlations for the remaining six ROIs (*rs* < 0.84, *ps* > 0.07).

#### 6-Month Female Mice pTau ICA

For pTau raw densities of 6-month female mice, two ICA identified sources led to a significant correlation between a weighted combination of brain region data and spatial reorientation performance. Source 1 from the 1-source iteration was positively correlated with spatial reorientation performance for 1.5 and 2.0 s difficulty levels (*rs*>0.95, ps≤0.01). In addition, source 1 from the 5-source iteration was positively correlated with the 1.5 s difficulty level (*r* = 0.996, *p* < 0.001; but not 2.0 s: *r* = 0.90, *p* = 0.17). The correlations between spatial reorientation performance and individual brain region correlations tended to be highest for the same regions that received a high weight for brain weighting profile that produced the significant correlations with spatial reorientation performance (e.g., for both r-values and ICA weights Sub was highest and RSP and CA1 were moderately correlated/weighted; Figure 7A). The remaining number of source iterations did not lead to any significant correlations with spatial reorientation ability for either difficulty level (1.5 or 2.0 s): 2 sources (*rs* < 0.77, *ps* > 0.12), 3 sources (*rs* < 0.71, *ps* > 0.18), 4 sources (*rs* < 0.82, *ps* > 0.09), 6 sources (*rs* < 0.96, *ps* > 0.06), 7 sources (*rs* < 0.82, *ps* > 0.09), and the remaining sources for the 5-source iteration (*rs* < 0.89, *ps* > 0.08).

#### 3-Month Female Mice pTau Individual Brain Region Correlations

First, we performed individual correlations between pTau positive cell density for each brain region and spatial re-orientation performance. Consistent with previous work linking dorsal structures to spatial navigation, a dorsal structure was the only structure to be significantly correlated with behavior. Higher density of pTau positive cells in dCA1 was associated with better spatial re-orientation performance for the 2.0 s difficulty level (Figure 7B, *Left*; less slowing in the reward zone; *r* = −0.997, *p* = 0.002) but not the 1.5 s difficulty level (*r* = −0.82, *p* = 0.18). There were a few other large but non-significant negative correlations (e.g., PC, and Sub; *rs* < 0.98, *ps* > 0.02; Bonferroni corrected critical *p* = 0.007). Surprisingly, unlike 6-month female mice where all correlations were positive (meaning more intracellular pathology was associated with worse performance), in 3-month female mice the correlations were negative (meaning that higher pTau positive cell density was associated with better performance).

#### 3-Month Female Mice pTau ICA

For pTau raw cell densities across brain regions, two ICA identified sources led to a significant correlation with spatial reorientation performance. Source 2 from the 5-source iteration was positively correlated with spatial reorientation performance for the 1.5 s (rs = −0.99, *p* = 0.04), but not the 2.0 s (*r* = −0.88, *p* = 0.12), difficulty level. In addition, source 6 from the 7-source iteration was positively correlated with spatial reorientation performance for the 1.5 s (rs = −0.99, *p* = 0.01), but not the 2.0 s difficulty level (*r* = −0.84, *p* = 0.16). The correlations between spatial re-orientation performance and individual brain region were highest for some of the same regions that received a high ICA weight for the source that produced this significant correlation with spatial re-orientation performance (e.g., both ICA weights and r-values were high for dCA1 and dSub) but differed substantially for other regions (Figure 7B, *Bottom*; PC is moderately correlated but received the lowest ICA weight). For pTau raw densities, the remaining number of source iterations did not lead to any significant correlations with spatial reorientation ability for either difficulty level (1.5 or 2.0 s): 1 source (*rs* > −0.35, *ps* > 0.65), 2 sources (*rs* > −0.88, *ps* > 0.12), 3 sources (*rs* > −0.98, *ps* > 0.05), 4 sources (*rs* > −0.96, *ps* > 0.15), 6 sources (*rs* > −0.94, *ps* > 0.06), the 7 sources iteration (*rs* > −0.88, *ps* > 0.12), and the remaining sources for the 5-source iteration (*rs* > −0.90, *ps* > 0.09).

#### 6-Month Male Mice pTau Individual Brain Region Correlations

In contrast to 6-month females, the correlation between dSub and spatial reorientation performance was low. Further, the largest correlations were a mixture of dorsal and ventral regions, though none were significant after Bonferroni correction (*rs* > −0.91, *ps* > 0.03, adjusted critical *p* = 0.007).

#### 6-Month Male Mice pTau ICA

For pTau raw cell densities across brain regions of 6-month male mice, one ICA identified source led to a significant correlation between raw pTau density and behavior data. Source 4 from the 6-source iteration was negatively correlated with spatial reorientation performance for the 2.0 s (*r* = −0.96, *p* < 0.05), but not the 1.5 s (*r* = −0.93, *p* = 0.10), difficulty level. Surprisingly, unlike 6-month female mice where all correlations were positive (meaning more intracellular pathology was associated with worse performance), in male mice the significant ICA correlation was negative (meaning that higher pTau positive cell density was associated with better performance). The correlations between spatial reorientation performance and individual brain regions were highest for some of the same regions that received a high ICA weight for the source that produced this significant correlation with spatial reorientation performance (e.g., both r-values and ICA weights are high for vCA1 and PC) but differed substantially for other regions (RSPd and dSub were moderately correlated but received high ICA weights; Figure 7C). For raw pTau densities, the remaining number of source iterations did not lead to any significant correlations with spatial reorientation ability for either difficulty level (1.5 or 2.0 s): 1 source (*rs*>−0.03, *ps* > 0.96), 2 sources (*rs* > −0.42, *ps* > 0.48), 3 sources (*rs* > −0.85, *ps* > 0.07), 4 sources (*rs* > −0.92, *ps* > 0.09), 5-sources (*rs* > −0.79, *ps* > 0.11), 7 sources (*rs* > 0.90, *ps* > 0.11), and the remaining sources for the 6 source iteration (*rs* > −0.64, *ps* > 0.24).

#### 12-Month Male Mice pTau Individual Brain Region Correlations

Next, we performed individual correlations between pTau positive cell density for each brain region and spatial re-orientation performance. Dorsal structures tended to have the strongest correlation values, but none were statistically significant (*rs* > −0.85, *ps* > 0.06).

#### 12-Month Male Mice pTau ICA

For pTau raw cell densities across brain regions, one ICA identified source led to a significant correlation with spatial reorientation performance. Source 4 from the 7-source iteration was positively correlated with spatial reorientation performance for the 1.5 s (rs = −0.97, *p* = 0.03), but not the 2.0 s difficulty level (*r* = 0.03, *p* = 0.97). So, similar to 6-month male mice higher pTau positive cell density was associated with better performance. The correlations between spatial reorientation performance and individual brain regions were highest for some of the same regions that received a high ICA weight for the source with a significant correlation with spatial re-orientation performance (e.g., both r-values and ICA weights were relatively high for RSPv, PC, and vSub) but differed substantially for other regions (Figure 7D; RSPd was moderately correlated but received the highest linear combination weight). For pTau densities, the remaining number of source iterations did not lead to any significant correlations with spatial reorientation ability for either difficulty level (1.5 or 2.0 s): 1 source (*rs* > −0.55, *ps* > 0.33), 2 sources (*rs* > −0.74, *ps* > 0.15), 3 sources (*rs* < 0.56, *ps* > 0.32), 4 sources (*rs* > −0.84, *ps* > 0.07), 5 sources (*rs* > −0.91, *ps* > 0.07), 6 sources (*rs* < 0.83, *ps* > 0.08), and the remaining sources for the 7-source iteration (*rs* < 0.88, *ps* > 0.13).

### 6E10 Positive Cell Density Profile Across Brain Regions Was Not a Significant Predictor of Spatial Re-orientation Performance

#### 6-Month Female Mice

Next, we performed individual correlations between raw 6E10 positive cell densities for each brain region and spatial re-orientation performance for 1.5 and 2.0 s difficulty levels. No single brain region was significantly correlated with spatial reorientation performance (*rs* > −0.79, *ps* > 0.11). For raw 6E10 cell densities across brain regions of 6-month female mice, no ICA results were significant (*rs* > −0.91, *ps* > 0.06).

#### 3-Month Female Mice

For 3-month female mice, as with 6-month male and female mice, the dSub region, but no other region, was significantly correlated with spatial reorientation performance for the 2.0 s difficulty level before, but not after correction for repeated testing (*rs* > −0.96, *ps* > 0.01, Bonferroni corrected critical *p* = 0.007). For raw 6E10 cell densities across brain regions of 3-month female mice, none of the ICA sources were significantly correlated with spatial reorientation ability for either difficulty level (1.5 or 2.0 s; *rs* > −0.93, *ps* > 0.06).

#### 6-Month Male Mice

For 6-month male mice, as with 6E10 data for 6-month female mice, no single brain region was significantly correlated with spatial reorientation performance (*rs* > −0.59, *ps* > 0.29). For 6E10 cell densities across brain regions of 6-month male mice, no ICA results were significant (*rs* > −0.96, *ps* > 0.06).

#### 12-Month Male Mice

For 12-month female mice, individual brain region correlations were notably different from the other three groups. Now cortical structures in the parietal-hippocampal network (RSC and PC) have the highest correlations and the correlations are mostly positive. The highest correlation was for RSCv for the 1.5 s difficulty level which was not significant after correcting for repeated testing (*r* = 0.93, *p* = 0.02, Bonferroni corrected critical *p* = 0.007) and none of the remaining 11 region/difficulty level combinations were significantly correlated with spatial reorientation performance (*rs* < 0.62, *ps* > 0.27). For 6E10 raw cell densities across brain regions of 12-month male mice, none of the ICA number of source iterations were significantly correlated with spatial reorientation ability for either difficulty level (1.5 or 2.0 s; *rs* < 0.82, *ps* > 0.08).

### Thioflavin S Staining Revealed a Low Number of Plaques in 12-Month Males but No Other Group

**Figure 8:**
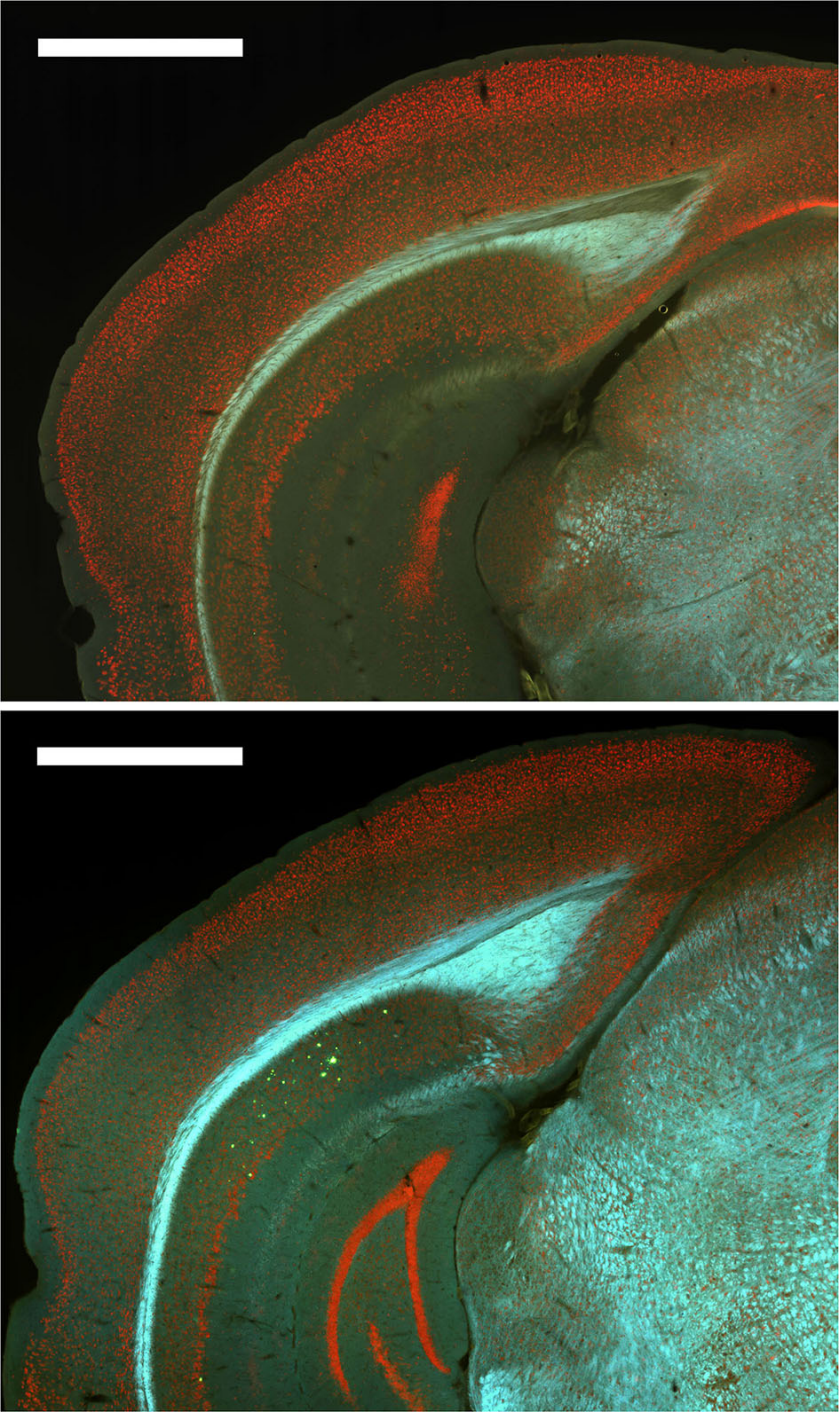
Plaques were absent from most brain regions for all groups except 12-month male 3xTg-AD mice. *Top*. Representative image of Thioflavin S staining for a 6-month female 3xTg-AD mouse with no plaques. Bottom. Representative image of Thioflavin S staining for a 12-month male 3xTg-AD mouse with plaques (green) in dorsal subiculum (dSub).

Finally, subjective examination of Thioflavin S staining of a subset of the mice revealed that plaques were only present in 12-month male mice and at relatively low levels (Figure 8). Therefore, no quantitative analyses were conducted with Thioflavin S staining.

## Discussion

We have previously shown that 6-month female 3xTg-AD mice were impaired at a spatial re-orientation task that requires the use of distal cues to become re-oriented, compared to non-Tg mice. In contrast, younger female mice (3-months) and male mice (6- and 12-month) were not impaired at spatial re-orientation. The impairment in 6-month female mice occurred early when tau and Aβ accumulation was low and prior to the emergence of extracellular pathology. We found, consistent with the exclusive impairment in 3xTg-AD 6-month female mice, only 6-month female mice had an ICA identified pattern of tau pathology across the parietal-hippocampal network that was positively correlated with behavior. Specifically, a *higher density of pTau positive cells* predicted *worse spatial learning and memory*. Within this network, subiculum stood out by receiving the highest ICA and CCA weights and as the only *individual* brain region that was significantly correlated with behavior. Surprisingly, despite a lack of impairment relative to controls, 3-month female and 6- and 12- month male mice also have patterns of tau pathology across the parietal-hippocampal brain network that were predictive of spatial learning and memory performance. However, the direction of the effect was opposite, a negative correlation, meaning that a *higher density of pTau positive cells* predicted *better spatial learning and memory*. Finally, though CCA analyses identified patterns of tau and 6E10 staining that were significant predictors of spatial learning and memory, ICA was only able to identify patterns of pTau staining across brain regions that were significant predictors of performance. Thus, the pattern of pTau positive cell density across the parietal-hippocampal network is a powerful predictor of spatial re-orientation learning and memory performance.

Our finding that a profile of pathology levels across brain regions as identified by CCA and ICA approaches is a better predictor of spatial reorientation deficits than single brain regions considered individually is consistent with a growing body of evidence suggesting that impairments observed in AD are a consequence of changes at the level of brain networks as opposed to individual brain regions (Yong et al., 2009; Zhao et al., 2012; Muñoz-Moreno et al., 2018). The spatial reorientation deficit we observed could explain the cause of spatial disorientation which is one of the first symptoms in humans with AD (Coughlan et al., 2018). Further, spatial navigation tasks like the one employed here can easily be adapted for humans as has been done with a variety of spatial navigation learning and memory tasks (e.g., Puthusseryppady et al., 2020; Mcavan et al., 2021). Our approach for identifying these brain network profiles is unique. Particularly with the ICA approach, which identifies patterns of staining across brain regions that are consistent across mice independent of behavior. We then asked if any of these independent components were predictive of spatial reorientation learning and memory. These tools have been previously applied to imaging data sets in humans (Mckeown et al., 2003; Wang et al., 2020; Zhuang et al., 2020) and could be applied to studying the progression from MCI to AD in humans performing spatial navigation tasks like the one employed here. Future studies could use this same approach to examine later timepoints in 3xTg-AD mice when tau and Aβ accumulation begins to follow an extracellular pathology progression profile that is better connected to what has been observed in humans (Mastrangelo and Bowers, 2008; Belfiore et al., 2019).

Our finding that tau accumulation in the parietal-hippocampal network is a better predictor of spatial reorientation performance than 6E10 is consistent with evidence suggesting that the level of tau accumulation is a better predictor of cognitive impairments than measures of amyloidosis (e.g., Lin et al., 2009). However, these studies have focused on later timepoints in disease progression. Here we show that this difference is apparent even very early, well before extracellular pathology is apparent. Further, ICA, which seeks independent brain network pathology patterns independent of behavior, was not able to identify any independent components from 6E10 data that were significant predictors of behavioral performance; however, CCA analysis, which uses behavioral data to explicitly seek a brain network profile that was associated with behavioral performance, was able to find a 6E10 brain network profile that was predictive of behavioral performance. It should also be noted that 6E10 is a non-specific stain (Aβ 1–16). In fact, we found that 6E10 staining was negatively correlated with M78 staining for all brain regions except hippocampus, potentially a consequence of this non-specific staining. Thus, while the M78 stain we used here (Aβ 1-42 fibrils) did not vary significantly across groups, it is possible that other more specific Aβ stains would have revealed a different pattern of results. Further, we previously found a correlation between 6E10 positive cell density in hippocampus and dysfunction in hippocampal-cortical interactions in 6-month female 3xTg-AD mice (Cushing et al., 2020), consistent with other work showing a similar relationship between 6E10 and dysfunctional LTP (Oddo et al., 2003). We also previously found that, though 6E10 levels in hippocampus were not directly predictive of spatial reorientation learning and memory performance, they did predict functional brain changes and these functional brain changes in turn predicted future spatial re-orientation learning and memory performance (Cushing et al., 2020). Finally, manipulations which remove Aβ in mice improve cognition (Billings et al., 2005; Iaccarino et al., 2016; Martorell et al., 2019). Together this previous work combined with the present findings suggest that while tau levels were the strongest predictor, it is very likely also true that Aβ was playing a role in the deficits in 6-month female 3xTg-AD mice.

Interestingly, differences between 6-month male and female 6E10 densities were only significant in the dorsal, but not ventral hippocampus. Dorsal hippocampus has been linked to spatial navigation, particularly for the spatial re-orientation task used here (Moser et al., 1993, 1995; De Hoz et al., 2003; Rosenzweig et al., 2003; Pothuizen et al., 2004). Since early spatial navigation deficits appear in humans with AD, this could suggest a potential neural correlate between 3xTg-AD mice and humans. It is possible that impaired formation of new proteins and dendritic spines may be leading to alterations in neural function, since these processes are important in memory and have been shown to be affected by Aβ (Balducci et al., 2010; Sadigh-Eteghad et al., 2015; Baglietto-Vargas et al., 2018). At the receptor level, Aβ can lead to changes in neural function by impacting a variety of receptor types (Sadigh-Eteghad et al., 2015). In particular, Aβ can interfere with glutamatergic receptors, which could lead to microglia-mediated synapse loss due to the recruitment of excess microglia (Rajendran and Paolicelli, 2018). However, ICA did not identify any 6E10 independent components that were significant predictors of spatial learning and memory performance for any of the groups of mice, but did for pTau for mice in each of the four groups. This suggests that tau accumulation, even pre-tangle, may be a stronger driver of dysfunction than amyloidosis. Consistent with this finding, Khan et al. (2013) showed that dysfunction in the PC can occur despite low levels of pathology in the PC as a consequence of tau pathology in entorhinal cortex and hippocampus, suggesting tau has widespread effects on network dynamics within the parietal-hippocampal network. Thus, there are likely multiple mechanisms driving the effects we observed which are likely a product of network level dysfunction.

ICA results that were significantly correlated with behavior, on average weighted the dSub contribution higher than any other brain region and dSub was one of the only individual brain regions that was significantly correlated with behavioral performance, despite pTau densities that were lower than 6 of the 7 other brain regions. Sub is one of the major outputs of CA1 and entorhinal cortex, and passes information to cortical areas, including RSC (Kitanishi et al., 2021). Previous research has shown that Sub can be partitioned into two regions, distal Sub and proximal Sub, which have been shown to be structurally and functionally different. In fact, distal Sub was shown to be necessary for a spatial task requiring the use of global cues, not local cues, in order to find an escape platform location, while it has been proposed that proximal Sub may be the region necessary for using local cues (Cembrowski et al., 2018). Sub regions have been shown to contain cell types necessary for spatial mapping such as grid cells, head direction cells, and boundary cells (Lever et al., 2009; Boccara et al., 2010; Epstein et al., 2017). More specifically, grid cells have been found in dorsal pre- and parasubiculum and these grid cells are colocalized with both head-direction and border cells. In addition, these cells can be modulated by head direction cells (Boccara et al., 2010). Here we examined subiculum proper, but future work should look at these subfields. Interestingly, Sub neurons have been shown to represent spatial information, such as place, speed, and trajectory, as accurately, or even more so, than CA1. An important role of Sub may be to create noise-resistant, high-firing rate, accurate representations for multiple types of navigational information (Kitanishi et al., 2021). Thus, Tau accumulation in Sub may be a critical driver of early hippocampal-parietal network dysfunction leading to the emergence of impaired spatial navigation in AD.

There are several observations about male mice that may give some hints about why even 12-month male mice that have some emerging extracellular pathology (i.e., a small number of plaques) are not impaired at spatial reorientation learning and memory, while 6-month female mice are impaired. First, male mice have lower M78 positive cell density in comparison to 6E10 positive cell density, which is significantly higher for 12-months males than all other groups. Given that M78 is thought to represent a more pathological conformation of Aβ, this difference could explain the absence of a spatial learning and memory deficit in male mice (Pensalfini et al., 2014). Second, 6- and 12- month 3xTg-AD male mice resemble 3-month female mice (but not 6-month female mice) in that there is a negative correlation between pTau density and behavior (meaning higher pathology density is associated with better spatial reorientation learning and memory).

Our results extend previous findings about the relationship between Aβ, tau, and cognitive impairments in early stages of tau and Aβ accumulation, and they also suggest that, at these early timepoints, the relationship between pathology and cognition is complex. For example, though 6-month females had the expected relationship between pathology and spatial learning and memory (mice with more tau accumulation performed worse), for younger female mice and all male mice the relationship was opposite (more tau accumulation was associated with better performance). Consistent with our findings, some have reported positive correlations with cognitive performance and pTau (more pathology is associated with better performance) for some tasks, time points in tau accumulation or with certain pTau epitopes (Huber et al., 2018). The presence of a similar positive correlation in males is especially complex given that the older 12-month male mice have started to develop some plaques. The absence of positive correlations in males could suggest that even though extracellular pathology is apparent, either some aspects of tau pathology may be less progressed in male mice or consequences of the pathology are altered as a function of sex (e.g., Park et al., 2007). Consistent with this idea, male mice (like 3-month female mice) were not impaired at spatial learning and memory; only 6-month female mice had impaired spatial learning and memory (Stimmell et al., 2019). Alternatively, correlations in young female mice may represent a transient, though likely complex, beneficial effect of low levels of tau accumulation that reverses as the mice age leading to the opposite direction of the correlation (more pathology associated with worse performance) in 6-month female mice. One potential explanation for our and other results showing a paradoxical relationship between tau and cognition may lie in the normal function of tau. There is increased phosphorylation of tau species induced by brain derived neurotrophic factor (BDNF) particularly in the hippocampus during stages of early development (including the Thyr202 site recognized by the AT8 tau antibody used here), which is likely related to increased neuronal plasticity during periods of prolific synaptogenesis (Goedert et al., 1989; Kosik et al., 1989; Hanger et al., 2009; Chen et al., 2012). Consistent with this hypothesis, reduced plasticity is seen with decreased tau phosphorylation (Sennvik et al., 2007; Hanger et al., 2009). Thus, in younger female mice a development-mediated role of phosphorylated tau in promoting hippocampal plasticity may explain the relationship between pTau levels and spatial learning and memory which is not present in the more mature, female, 6-month AD mice leading to the opposite relationship with spatial learning at that age. Consistent with this hypothesis, hippocampus received the highest ICA weights in 3-month female mice.

In summary, we previously showed impairments in spatial re-orientation in 6-month female 3xTg-AD mice early when levels of tau and Aβ accumulation are low. Here we show that 6-month female mice have an ICA identified pattern of intraneuronal pTau (but not 6E10) density across the parietal-hippocampal network that is a significant predictor of spatial re-orientation impairments. Surprisingly, similar patterns were identified in 3-month female mice, prior to the emergence of a spatial learning and memory deficit (and also 6- and 12-month male mice with intact spatial learning and memory), that were also predictive of behavioral performance. However, in 3-month female mice (and 6- and 12-month male mice) the relationship was opposite that observed in 6-month female mice so that higher density of pTau positive cells is associated with better performance. This suggests that early tau accumulation, while potentially a strong driver of impaired spatial re-orientation, is also a complex and dynamic process.

## Data Availability Statement

The raw data supporting the conclusions of this article will be made available by the authors, without undue reservation.

## Ethics Statement

The study was reviewed and approved by Florida State University Animal Care and Use Committee.

## Author Contributions

AW designed the experiment. AS, SM, SC, and AW with assistance from DF, JD, LS-M, RA, CG-B, and SR conducted the experiment. WW and ZX assisted with data analysis design. AS, ZX, JD, and AW analyzed the data and wrote the manuscript including figures. All authors reviewed the manuscript.

## Conflict of Interest

The authors declare that the research was conducted in the absence of any commercial or financial relationships that could be construed as a potential conflict of interest.
